# The complete mitochondrial genome of *Syritta pipiens* (Linnaeus, 1758) (Diptera: Syrphidae) and phylogenetic analysis

**DOI:** 10.1080/23802359.2021.1957035

**Published:** 2021-07-26

**Authors:** Ya-Qin Shi, Juan Li, Hu Li

**Affiliations:** Shaanxi Key Laboratory of Bio-resources, School of Biological Science & Engineering, Shaanxi University of Technology, Hanzhong, China

**Keywords:** Hoverfly, *Syritta*, mitogenome, phylogeny

## Abstract

The complete mitochondrial genome (mitogenome) of *Syritta pipiens* (Linnaeus, 1758) was sequenced with 15,745 bp in length including 37 genes and a non-coding region. The overall nucleotide composition showed a strong AT bias. Most protein-coding genes (PCGs) used ATN as the start codon while *ATP6* and *ND1* used TTG, and stopped by TAA or TAG but *ND5* ended with an incomplete T. Phylogenetic trees were reconstructed based on the 24 complete mitochondrial sequences from Syrphidae using the methods of maximum-likelihood (ML) and Bayesian inference (BI), resulted in *S. pipiens* clustered into the clade of Eristalinae, which conformed to the traditional classification, but the trees did not support the monophyly of Eristalinae. More molecular data is needed for further study.

*Syritta pipiens* (Linnaeus, 1758), the ‘thick-legged hoverfly’, has an enlarged hind femur, and the abdomen with three pairs of yellow spots, it is distributed in the Palearctic (widespread in Europe), Nearctic and Oriental regions (Pérez-Bañón and Marcos-García 2000). Adult of *S. pipiens* visits flowers of wide range; they usually occur in the autumn, and largely flying around the Ivy flowers (Ball and Morris 2015). The taxonomic status of *S. pipiens* has been argued, it is classified into the tribe Milesiini in the work of Huang and Cheng (2012), whereas into Xylotini in the work of Ball and Morris (2015). Until now, the study of the complete sequence of the mitochondrial genome of *Syritta* has not been reported, that *S. pipiens* represents the genus *Syritta* in this article for the first time to be sequenced with the mitogenome.

Using the mitochondrial genomes (mitogenomes) data to rebuild and discuss the phylogenetic relationship of invertebrates is widely accepted by many authors (Cameron et al. 2007; Cameron 2014; Li 2019; Wang et al. 2019). It is a dependable method due to the mitogenome with features of maternal inheritance, stable gene composition, relative gene sequence conservation, and minimal recombination (Cameron et al. 2007; Lavrov 2007). To date, there are 23 species of Syrphidae have been registered in GenBank (https://www.ncbi.nlm.nih.gov/). In this study, we provide a new complete mitogenome of *S. pipiens* (Genbank: MN494095).

Adult specimens were collected by sweep net in Changqing National Nature Reserve (107°55′E, 33°60′N), Shaanxi Province, China. Collector: Hu Li, lihu@snut.edu.cn. Voucher specimens were deposited in Shaanxi Key Laboratory of Bio-resources, Shaanxi University of Technology, Hanzhong, China (SUHC) (accession number of the specimen for sequencing in this study is 201902-32). Genomic DNA was extracted by a TIANamp Genomic DNA Kit (Tiangen, Beijing, China), according to manufacturer’s protocol, except slightly modified by, specimens being placed in a water bath at 56 °C for 8 h to ensure cell lysis. The sample was sequenced in an Illumina NovaSeq6000 platform, the sequencing mode was 150PE.

The complete mitogenome was assembled using Geneious prime (v2019 1.3) (Kearse et al. 2012). The secondary structure and position of 22 transfer RNA genes (tRNAs) were predicted by ARWEN (v1.2) (Laslett and Canbäck 2008) and checked manually. The 13 protein-coding genes (PCGs) were annotated by finding the opening reading frame with the invertebrate mitochondrial genetic code in Geneious prime (v2019 1.3) (Kearse et al. 2012). The positions of *16S rRNA* and *12S rRNA* were determined by homologous comparison with other closely related species. The control region was located between *12S rRNA* and *tRNA-Ile*.

The total length of the complete mitogenome of *S. pipiens* was 15,745 bp with 37 genes, including 13 PCGs, 22 tRNAs, 2 rRNAs, and a non-coding region. All 37 typical mitochondrial genes were congruent with ancestor positions and directions in terms of gene arrangement (Clary and Wolstenholme 1985). In this complete mitogenome, A + T content was 78.8% (A: 40.4%, T: 38.4%, G: 8.8%, C: 12.4%). For start codons, most PCGs started with standard codon ATN, except for *ATP6* and *ND1* that initiated with TTG. 12 genes terminated with TAA or TAG, *ND5* ended with an incomplete T; such incomplete termination codons are common in the mitogenomes of metazoan animals, and it is speculated that they may be formed after transcription through the action of poly-adenylate to complete termination of transcription (Ojala et al. 1981). Most of the secondary structures of tRNA are of cloverleaf structure, except *tRNA-Ser ^AGC^* lacks DHU arm.

We reconstructed the phylogenetic trees based on 13PCGs and 2rRNAs from 24 Syrphidae species and 2 outgroup species (Figure 1). Using TranslatorX online server (http://161.111.161.41/index_v4.html) (Abascal et al. 2010) and MAFFT v7 online server (https://mafft.cbrc.jp/alignment/server/) (Katoh et al. 2019) for 13 PCGs and 2 rRNAs sequences alignment, respectively. The best partitioning schemes and substitution models were determined with PartitionFinder2 on XSEDE (2.1.1) using Akaike Information Criterion (AIC) (Lanfear et al. 2017) (https://www.phylo.org/portal2/task!list.action). Bayesian inference (BI) and maximum likelihood (ML) analysis were respectively performed using MrBayes (v.3.2.7) within the CIPRES webserver (https://www.phylo.org/portal2/login!input.action) and IQ-TREE webserver (http://iqtree.cibiv.univie.ac.at/) (Trifinopoulos et al. 2016). Both topologies of BI and ML trees support the monophyly of Syrphinae, which agrees with the study of Young et al. (2016). Meanwhile, the phylogenetic trees showed that *S. pipiens* was clustered in the tribe Eristalini clade, and does not congruent with traditional taxonomy. So Further investigation will be needed to obtain more complete mitogenomes of representative species especially the species of Xylotini and Milesiini to discuss the classification status of *S. pipiens*.

**Figure 1.**
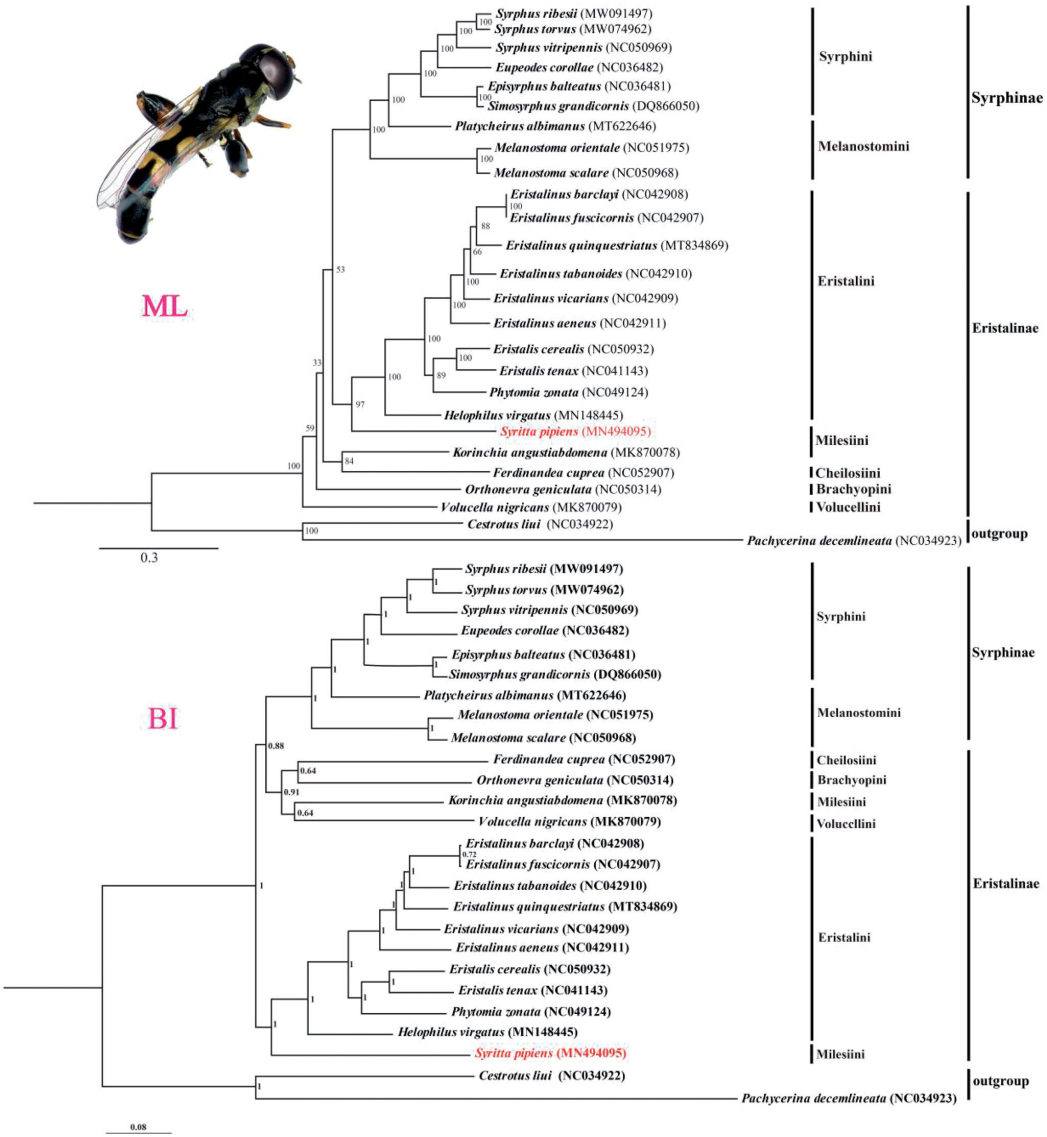
Maximum-likelihood (ML) and Bayesian inference (BI) phylogenetic trees based on the concatenated sequences of 13PCGs and 2rRNAs genes from mitochondrial genome of 24 Syrphidae species and two outgroups. The numbers of branches indicate bootstrap value. The adult imaging of *S. pipiens* is showed at upper-left corner.

## Data Availability

The data that support the findings of this study are openly available in GenBank at https://www.ncbi.nlm.nih.gov/genbank/, reference number MN494095.The associated SRA, BioProject and Bio-Sample numbers are SRR14453871, PRJNA727697 and SAMN19022671, respectively.
